# Effect of the Heaviness of Smoking Index (HSI) and Duration of Smoking on Central Neural Processing Using Brainstem Auditory Evoked Responses (BAERs)

**DOI:** 10.7759/cureus.68228

**Published:** 2024-08-30

**Authors:** Muskan Singh, Manjinder Kaur, Renu Khamesra, Umang Arora, Nimit Boonlia

**Affiliations:** 1 Physiology, Geetanjali Medical College and Hospital, Udaipur, IND; 2 Neurology, Geetanjali Medical College and Hospital, Udaipur, IND

**Keywords:** cigarette smoking, cortical excitability, acoustic evoked brainstem potentials, evoked potentials, tobacco use

## Abstract

Purpose of study

The goal of this research was to find the correlation of nicotine dependence and duration of smoking with the status of central neuronal processing in chronic smokers. Our primary objective was to record brainstem auditory evoked responses (BAERs) in chronic smokers and further find their correlation to the Heaviness of Smoking Index (HSI) scores and years of non-abstained smoking of the subjects. We postulated that smoking leads to myelination abnormalities which in turn causes decreased impulse conduction velocity.

Methods

After obtaining informed consent, we conducted BAER on 60 male smokers who were further classified into groups based on their HSI scores (low, moderate, and high nicotine dependency) and 20 age-matched, non-smoking males. The obtained data was examined using the two-way ANOVA test and the Kruskal-Wallis test. Pearson's coefficient of correlation and the median (as a measure of central tendency) were calculated.

Results

We observed a non-significant negative correlation between wave I BAER latency and the degree of nicotine dependence. Wave II showed minimal correlation, whereas a positive correlation was seen in waves III, IV, and V. Interpeak latencies (IPL) I-III and III-V showed a non-significant positive correlation with the HSI score, whereas IPL I-V showed a significant positive correlation with the same.

When correlated with the duration of smoking (years), the latencies (msec) of BAER waves I-V showed a pattern of progressively decreasing negative correlation, out of which waves I, II, and III were significantly affected. The IPL (msec) of waves I-III was non-significantly, yet positively, correlated, while the IPL of waves I-V and III-V showed a significant positive correlation to the duration of smoking.

Conclusions

The degree of nicotine dependence and duration of tobacco smoking progressively affected the latencies of BAER waves at the pontomedullary level of the brainstem. This indicates slower central neuronal processing at this level and an increased central transmission time, the extent of which is directly dependent on the extent of tobacco smoking. This is attributed to the myelination defects caused by direct and indirect effects of the toxic metabolites of tobacco smoke, chronic hypoxia, hypercapnia, and respiratory acidosis.

## Introduction

Tobacco smoking has become a global pandemic and is a leading cause of various disorders. These effects are mainly produced by the components of tobacco smoke, namely, nicotine and toluene [1], or due to the hypoxic and hypercapnic states caused by the inhaled smoke [2,3]. According to the World Health Organization (WHO), the global adult tobacco consumption prevalence in 2020 was 22.3%, with a distribution of 36.7% among men and 7.8% among women, causing eight million deaths worldwide in 2020 [4]. As medical science advances, new studies are published every day, shedding light on the newer aspects of the outcomes of tobacco smoking. Yet, a lacuna remains constant, with little research on the effect of tobacco smoking on the central nervous system (CNS).

It has been observed that tobacco smoking targets the cognitive domains of the human brain. This effect is caused because nicotine is a large component of tobacco smoke, which acts via binding to a vast number of nicotinic acetylcholine receptors (nAChRs) throughout the CNS (mainly subcortical structures) and hence influences the said areas of the brain [5].

If so, it is not a long shot to think that chronic exposure to nicotine might have a long-term impact on central neuronal processing. Some of the functions expected to be affected are somatosensory, motor, executive, learning, and memory [1].

Due to the known involvement of the sensory component of cognition and to measure the long-term effects of tobacco smoke on central neuronal processing, we opted to measure the brainstem auditory evoked responses (BAERs) in chronic tobacco smokers. BAER provides a non-invasive means of examining the auditory aspect of CNS functions and, hence, is a promising tool to measure the magnitude of neurological changes precipitated by long-term exposure to tobacco smoke.

Some of the previous studies have also shown a significant prolongation of latencies in waves I and III in chronic smokers. Researchers have observed significant subclinical abnormalities in the brainstem auditory evoked potentials, with a significant correlation to FEV1 and smoking pack-years [6]. This effect on the BAER latencies has been reported as prolongation of BAER wave III latency and interpeak latencies (IPL) I-III. Further, long-term smoking had been associated with chronic hypoxia and hypercapnia, which could potentially result in the prolongation of these waves [3]. Recently, studies have shown that chronic smoking results in a decline in cognitive functioning [1]. Further, the effect of tobacco smoking on the ulnar and median nerves delayed the conduction velocity in sensory nerve fibers due to peripheral demyelination or radiculopathy due to higher oxidative stress in a chronic smoker [7,8].

The existing literature clarifies the definite effect of tobacco smoke (either due to its components or hypoxia/hypercapnia caused by it) on the CNS and other sensory nerves in general. However, a study showing the correlation of the Heaviness of Smoking Index (HSI) and duration of smoking to the extent of changes in BAER recordings, with the intention to demonstrate the degree of the abovementioned effects, has not been conducted till date.

The present study aims to find the relationship between the HSI and duration of smoking on central neuronal processing, if any, via the BAERs in chronic smoker cases and age-matched, non-smoker controls.

## Materials and methods

Study design 

The study was designed as an observational, case-control study, which was started only after obtaining ethical clearance from the Human Research Ethics Committee of Geetanjali University on March 28, 2022 (approval number: GU/HREC/EC/2022/2021).

The participants were recruited by a simple random sampling method. 

Sample Size Calculation

A total of 73 participants are necessary to compare the four groups (non-smoker (NS), smoker with low nicotine dependence (SL), smoker with moderate nicotine dependence (SM), and smoker with high nicotine dependence (SH)) (as elaborated below) with a significance level (alpha) of 5%, a confidence index of 95%, a power of 80%, and a large effect size. To ensure a balanced ANOVA analysis, the sample size has been increased to 80 participants (20 per group), which provides a statistical power of 85% and accounts for potential losses during the data cleaning process. The final sample size is therefore set at 80 participants.

They were internally divided into Group S or the smoking group (cases) and Group N or the non-smoking group (controls). 

Group S (cases) comprised a total of 60 participants, fulfilling the inclusion criteria. They were further divided into three equal subgroups, namely, SL (HSI score: 0-2), SM (HSI score: 3-4), and SH (HSI score: 5-6), on the basis of the extent of nicotine dependence (determined by the HSI scores).

HSI is a smaller derivative of the Fagerstrom test for nicotine dependence (FTND) which depends on two conditions: "time to first cigarette upon waking" and "the quantity of cigarettes smoked in a day" [9]. The overall HSI score is calculated by adding together the points achieved across the factors mentioned previously.

Group N (controls) (NS) comprised 20 participants, cohering to the inclusion and exclusion criteria. 

Inclusion criteria 

Group S (Cases)

Male volunteers willing to give consent for participation in the study, between the ages of 18 (can provide informed consent independently) and 50 years (before the onset of geriatric changes), and with a history of three or more years of tobacco smoking without abstinence were recruited.

Group N (Controls)

Male volunteers willing to give consent for participation in the study, between the ages of 18 (can give informed consent independently) and 50 years (before the onset of geriatric changes), and with no history of prior tobacco smoking were recruited as well. 

Exclusion criteria

The study excludes individuals with specific medical histories and conditions, including chronic alcohol consumption, ongoing or previous pulmonary disease, smoking substances other than tobacco, history of substance abuse, chronic systemic diseases such as hypertension or diabetes mellitus, and generalized neurological disorders affecting neuronal processing. Additionally, participants with any cardiac pathology (such as ischemic heart disease, congestive heart failure, or valvular dysfunction), with current or past ear infections, with conductive or sensorineural hearing loss, or using hearing aids were excluded. Furthermore, individuals who did not consent to participate in the study were also excluded from participation.

Data collection

Setting

The study was conducted in the neurology department of a tertiary care hospital on the highly sophisticated Nihon Kohden z (DC-940BK, Japan) in a sound- and light-proof room.

Preparation 

The participant was asked to wash their hair on the day of the test. The hair was dry and non-oiled during the course of the test. Both ears of each participant were cleaned using cotton swabs prior to the conduction of the test. The age, height, weight, contact details, duration of smoking (in years) (without abstinence), and HSI score (for group S) were recorded in their respective case record proforma sheet before the test.

Procedure

**Figure 1:**
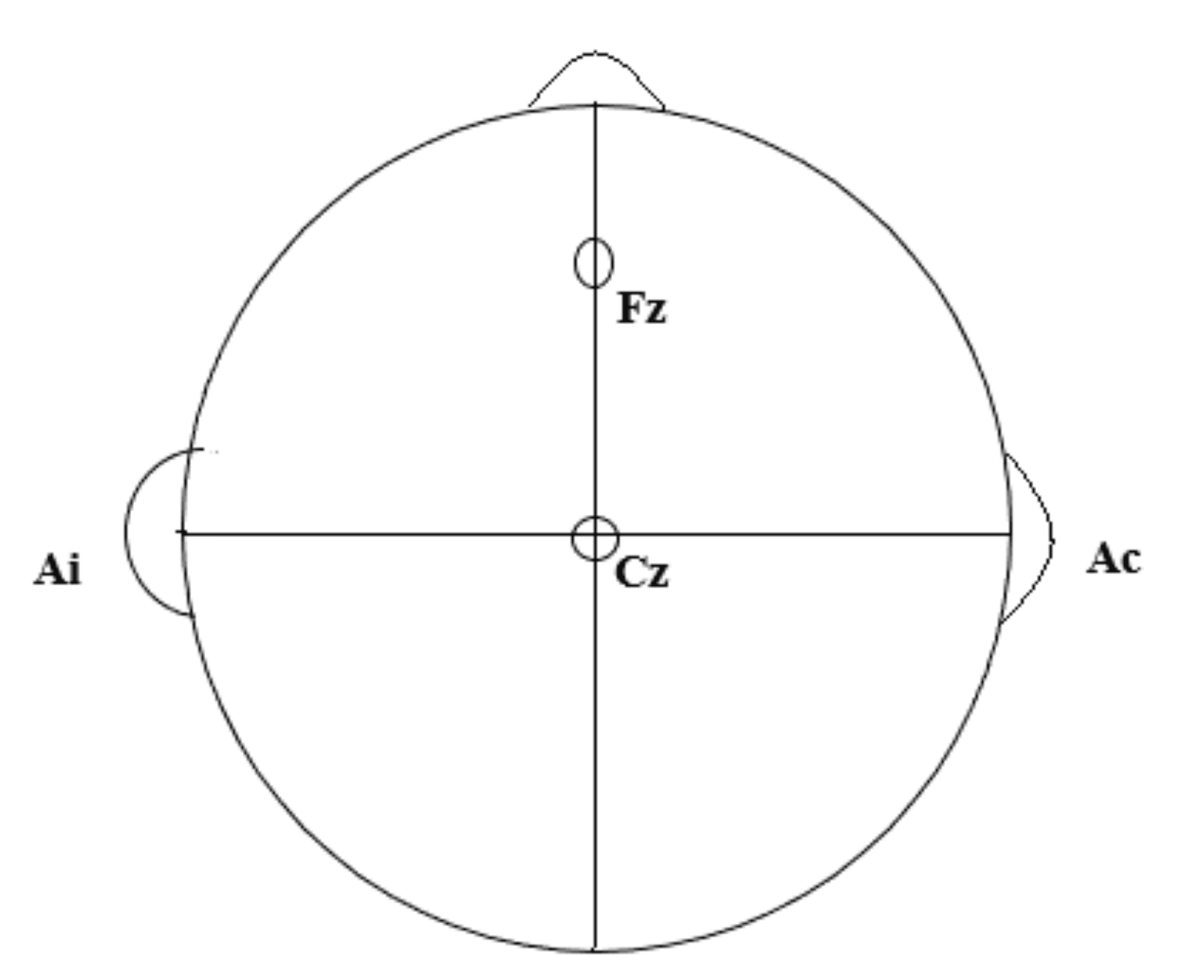
Electrode placement for the recording of BAER waves in the left ear BAER: brainstem auditory evoked response

The BAER was recorded using the 1-cm-diameter disc surface electrodes filled with conducting jelly [10]. They were placed at the mastoid processes of ipsilateral (Ai) and contralateral (Ac) ears and were referred to the Cz point on the vertex. The ground was placed on the Fz point (Figure 1). Two channels were formed by the abovementioned placement of electrodes, namely, channel 1 (Ai to Cz) and channel 2 (Ac to Cz). 

Stimulation

Each participant was made to wear a pair of noise-canceling headphones. A cotton plug was placed in the contralateral ear to produce a masking effect. BAERs were produced by brief click stimuli, which were square-wave pulses of 0.1 msec duration. These clicks were presented 10-30 times per second at an intensity of 60-70 Db using the noise-canceling earphones [10]. 

Recording

The BAER was recorded using an amplification of 200,000 Hz; the low filter was set at 100 Hz and the high filter at 3 kHz [10]. It was done in the rarefaction phase (movement of sound away from the patient's ear). The recording showed 5-8 vertex-positive peaks, which were labeled using Roman numerals. The initial five waves are of clinical interest, whereas the rest of the waves are highly variable and hence not relevant with respect to the study [10].

Interpretation

**Figure 2:**
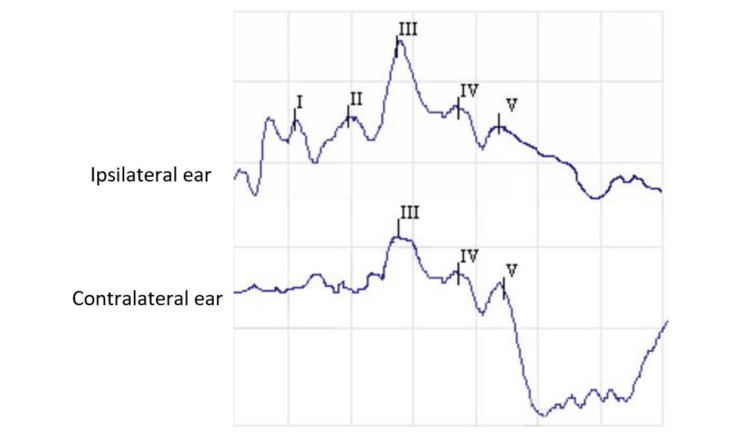
BAER waves as recorded from the left ear BAER: brainstem auditory evoked response

As illustrated in Figure 2, there are five waves. Wave I originates from the auditory nerve and appears as an initial upgoing peak in Ai, whereas it may be attenuated in the contralateral ear. It appears 1.4 msec after the stimulus [10]. Wave II originates from the cochlear nuclei and appears as a small peak along with the downgoing slope of wave I or the upgoing slope of wave III. It is prominent in the contralateral channel if wave I is absent [10]. Wave III begins from the superior olivary nuclei in the medulla oblongata and is seen as a prominent peak which appears smaller and earlier in the contralateral ear because its amplitude is similar at the vertex and contralateral ear [10]. Waves IV and V originate from the lateral lemniscus and the inferior colliculus of the midbrain, respectively. Wave V is the most prominent peak, appearing 5.5 msec after the stimulus. In the ipsilateral ear, wave IV fuses with V, resulting in the wave IV-V complex [10]. 

Measurements of BAER Parameters

Absolute latencies: These measurements were taken from the beginning of recording to the peaks of the individual waves, indicating the total time required for an impulse to reach the respective nuclei [10].

IPL: The commonest IPL employed in clinical practice are I-V, I-III, and III-V. IPL I-V is computed as the difference between the latencies of wave V and wave I. It is a measure of conduction time from the proximal VIII nerve (after the generation of impulse) to the midbrain (inferior colliculus). It is normally 4.5 msec [10]. IPL I-III is calculated as the latency difference between the peaks of wave III and wave I. It is a measure of conduction from the proximal VIII nerve (after impulse generation) to the lower pons (superior olivary nucleus). It is normally 2.5 msec [10]. IPL III-V measures the conduction time from the lower pons (superior olivary nucleus) to the midbrain (inferior colliculus). It is normally 2.4 msec [10].

Statistical analysis

The data analysis of all the groups was performed using Microsoft Excel Data Analysis ToolPak and IBM SPSS Statistics for Windows, Version 26.0 (Released 2019; IBM Corp., Armonk, New York, United States).

The median value was calculated as a measure of central tendency, and the interquartile range (IQR) was calculated as a measure of dispersion. The Kruskal-Wallis test for significance was applied to these values for all parameters, across each individual group.

Pearson's coefficient of correlation was further calculated to find any significant correlation between the HSI score and the latencies and IPL of the BAER waves. For this, the mean values of latencies and IPL for the left and right ears were calculated and used. The same procedure was used to find the correlation between the duration of smoking and the parameters of BAER waves.

Analysis of variance was done using a two-way ANOVA to assess any significance of difference in the recorded values of latencies and IPL of each BAER wave between the left ear and the right ear.

## Results

In Table 1, the median values of age, BMI, duration of smoking, and HSI scores all showed significant differences across the four groups, with p-values <0.001, 0.041, 0.009, and <0.001, respectively.

**Table 1:** Median values of personal attributes in the respective groups *: significant with p<0.05; BMI: body mass index; HSI: Heaviness of Smoking Index; NS: non-smoker; SL: smoker with low nicotine dependence; SM: smoker with moderate nicotine dependence; SH: smoker with high nicotine dependence

	Median				Kruskal-Wallis test (H)	df	P-value
	NS (n=20)	SL (n=20)	SM (n=20)	SH (n=20)			
Age (years)	21	21.5	34.5	24.5	10.018	3	<0.001*
BMI (kg/m^2^)	22.5	19.86	18.82	23.1	8.76	3	0.041*
Duration of smoking (years)	-	4	10	7	9.46	2	0.009*
HSI score	-	0	3	5.5	54.67	2	<0.001*

In Table 2, the median value of individual latencies (msec) was calculated for each group, and the Kruskal-Wallis test was applied to the data, which revealed no significance of difference for the values across all four groups.

**Table 2:** Latencies (msec) of the BAER waves I-V L: left ear; R: right ear; NS: non-smoker; SL: smoker with low nicotine dependence; SM: smoker with moderate nicotine dependence; SH: smoker with high nicotine dependence; BAER: brainstem auditory evoked response

Latencies (msec)	Median								Kruskal-Wallis test (H)		df	P-value	
	NS (n=20)		SL (n=20)		SM (n=20)		SH (n=20)						
	L	R	L	R	L	R	L	R	L	R		L	R
Wave I	1.365	1.345	1.27	1.36	0.92	1.37	1.19	1.185	5.986	3.852	3	0.148	0.371
Wave II	2.35	2.41	2.35	2.43	1.54	2.28	1.98	2.38	6.201	1.348	3	0.134	0.928
Wave III	3.455	3.51	3.38	3.405	3.24	3.53	3.41	3.16	7.945	4.584	3	0.061	0.274
Wave IV	4.655	4.625	4.34	4.71	3.85	4.38	4.62	4.305	5.905	5.123	3	0.153	0.217
Wave V	5.36	5.435	5.09	5.32	5.06	5.155	5.36	5.245	7.606	4.708	3	0.071	0.259

In Table 3, the median values of the IPL (msec) of waves I-III, I-V, and III-V were individually calculated. The Kruskal-Wallis test was applied. The result indicated a significant difference in IPL I-V across the four groups, for both left and right ears, with a p-value of 0.05 and 0.04, respectively.

**Table 3:** Interpeak latencies (msec) in BAER recording *: significant with p<0.05; L: left ear; R: right ear; NS: non-smoker; SL: smoker with low nicotine dependence; SM: smoker with moderate nicotine dependence; SH: smoker with high nicotine dependence; BAER: brainstem auditory evoked response

Interpeak latencies (msec)	Median								Kruskal-Wallis test (H)		df	P-value	
	NS (n=20)		SL (n=20)		SM (n=20)		SH (n=20)						
	L	R	L	R	L	R	L	R	L	R		L	R
Waves I-III	2.065	2.095	2.04	2.075	1.9	2.02	2.035	2.045	4.857	0.693	3	0.243	1
Waves I-V	4.03	4.13	3.89	3.935	3.91	3.795	4.315	3.81	8.358	8.879	3	0.05*	0.04*
Waves III-V	1.99	2.015	1.93	1.835	2.03	1.84	1.96	1.83	2.495	3.828	3	0.649	0.378

**Figure 3:**
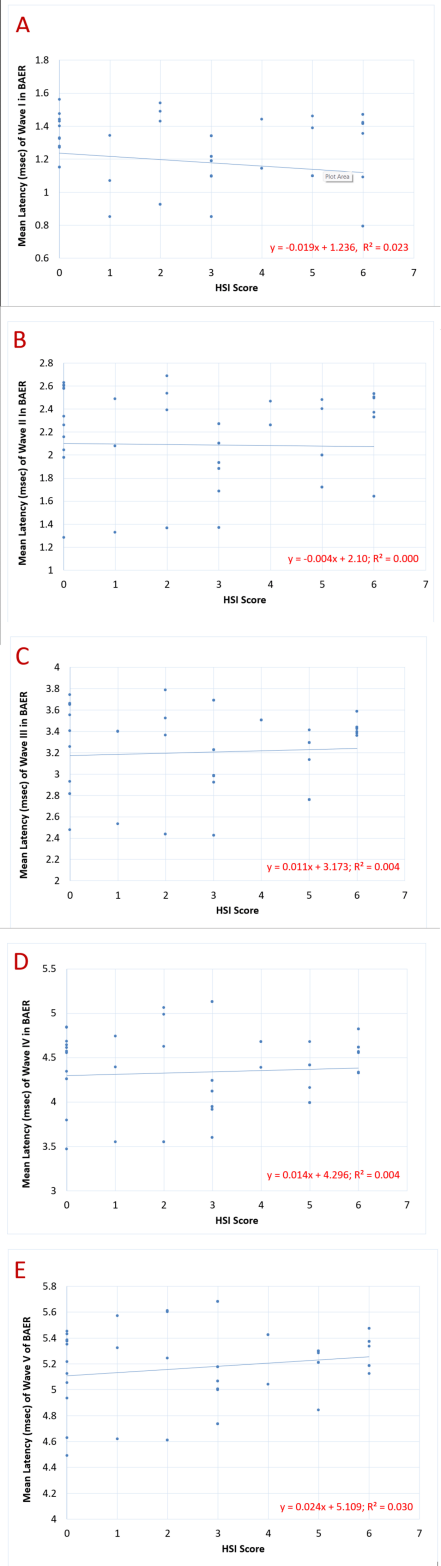
Correlation of HSI score with latency (msec) of BAER waves I-V in chronic smokers (n=60): (A) p=0.240 (non-significant), (B) p=0.852 (non-significant), (C) p=0.630 (non-significant), (D) p=0.602 (non-significant), and (E) p=0.179 (non-significant) HSI: Heaviness of Smoking Index; BAER: brainstem auditory evoked response

Figure 3 shows the correlation of the HSI score with the latencies (msec) of BAER waves I-V. The mean value of the latencies (msec) of both left and right ears was calculated for each wave, and tests for correlation were applied to the data. The results indicated a non-significant correlation of latencies (msec) of waves I, II, III, IV, and V with the HSI score.

**Figure 4:**
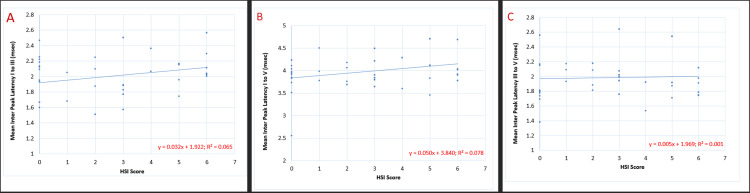
Correlation of HSI score with interpeak latencies (msec) of BAER waves I-III, I-V, and III-V in chronic smokers (n=60): (A) p=0.07 (non-significant), (B) p=0.03*, and (C) p=0.77 (non-significant) HSI: Heaviness of Smoking Index; BAER: brainstem auditory evoked response; *: significant with p<0.05

Figure 4 shows the correlation of the HSI score with the IPL (msec) of BAER waves I-III, waves I-V, and waves III-V. The mean value of the IPL (msec) of both left and right ears was calculated, and tests for correlation were applied to the data. The results indicated that IPL (msec) I-V showed a significant correlation with the HSI score (p-value 0.03), whereas that of I-III and III-V showed a non-significant correlation.

**Figure 5:**
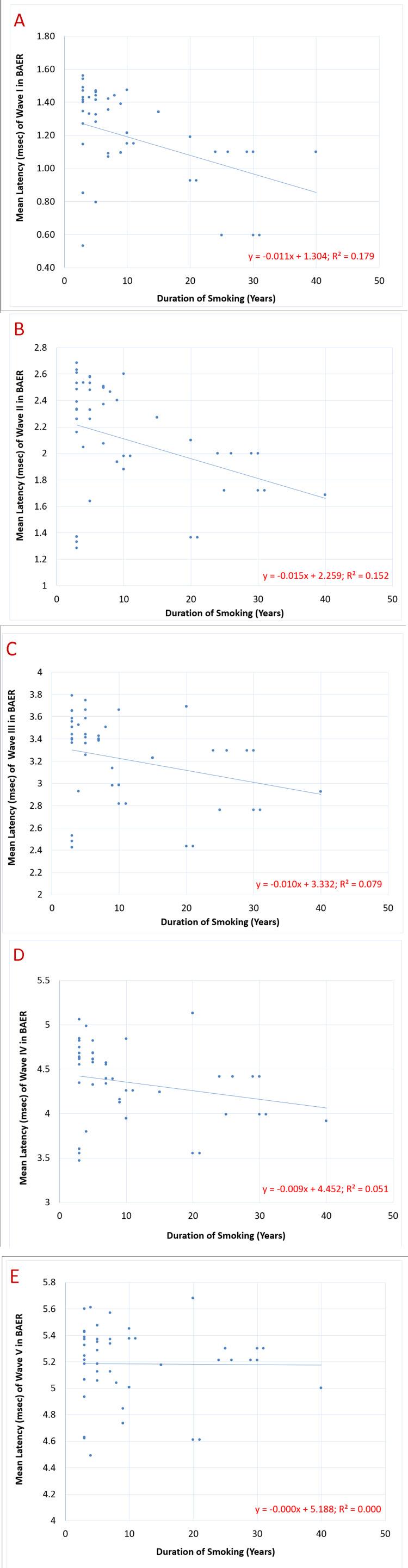
Correlation of the duration of smoking (years) with latencies (msec) of BAER waves I-V in chronic smokers (n=60): (A) p=0.00074**, (B) p=0.0020**, (C) p=0.029*, (D) p=0.08 (non-significant), and (E) p=0.93 (non-significant) BAER: brainstem auditory evoked response; *: significant with p<0.05; **: highly significant with p<0.01

Figure 5 shows the correlation of the duration of smoking (years) with the latencies (msec) of BAER waves I-V. The mean value of the latencies (msec) of both the left and right ears was calculated for each wave, and tests for correlation were applied to the data. The results indicated that the latencies (msec) of waves I, II, and III showed a significant negative correlation with the duration of smoking (years), with a p-value of 0.00074, 0.0020, and 0.029, respectively, whereas latencies of waves IV and V showed a non-significant negative correlation with the same.

**Figure 6:**
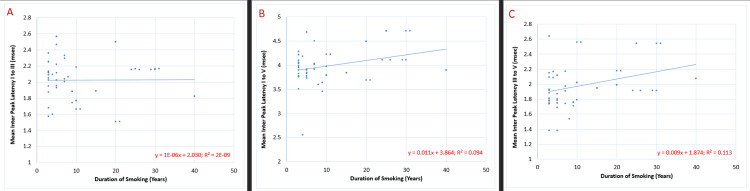
Correlation of the duration of smoking (years) with interpeak latencies (msec) of BAER waves I-III, I-V, and III-V in chronic smokers (n=60): (A) p=0.99 (non-significant), (B) p=0.017*, and (C) p=0.009** BAER: brainstem auditory evoked response; *: significant with p<0.05; **: highly significant with p<0.01

Figure 6 shows the correlation of the duration of smoking (years) with the IPL (msec) of BAER waves I-III, waves I-V, and waves III-V. The mean value of the IPL (msec) of both left and right ears was calculated, and tests for correlation were applied to the data. The results indicated that IPL I-V and III-V showed a significant positive correlation with the duration of smoking, with a p-value of 0.017 and 0.009, respectively, whereas the IPL I-III showed a non-significant positive correlation with the same.

Table 4 shows the variance in the extent of change in BAER latencies (msec) (waves I-V) between the left and right ears. A two-way ANOVA test was applied to the data of the left and right ear for each BAER wave, and the F-value and P-value were obtained. A significant p-value denotes that the extent of change in the latency of the respective BAER wave in the left and right ear is significantly different from each other. Results indicate that there is a significant variance in the latencies of waves I, III, and IV between the left and right ears, with a p-value of 0.02, 0.05, and 0.03, respectively, whereas no significant variance is seen in the latencies of waves II and V between the left and right ears.

**Table 4:** Variance in latencies (msec) of BAER waves I-V between the left and right ears by the two-way ANOVA in smokers (n=60) *: significant with p<0.05; BAER: brainstem auditory evoked response

Left and right ears	F	P-value
Wave I	3.19	0.02*
Wave II	1.10	0.35
Wave III	2.65	0.05*
Wave IV	3.08	0.03*
Wave V	1.87	0.14

Table 5 shows the variance in the extent of change in IPL (msec) of BAER between the left and right ears. A two-way ANOVA test was applied to the data of the left and right ear for each IPL, and the F-value and P-value were obtained. A significant p-value denotes that the extent of change in the respective IPL in the left and right ear is significantly different from each other. Results indicate no significant variance in IPL between the left and right ears.

**Table 5:** Variance in interpeak latencies (msec) of BAER waves between the left and right ears by the two-way ANOVA in smokers (n=60) BAER: brainstem auditory evoked response

Left and right ears	F	P-value
Waves I-III	1.61	0.19
Waves I-V	1.62	0.19
Waves III-V	1.12	0.34

## Discussion

According to the aforementioned information, the present study compared 20 non-smokers to 60 smokers with low, moderate, and high nicotine dependence. The BAER parameters discussed previously represent the progressive activation of different levels of the auditory pathway in the form of multiple waves, starting from the proximal part of the acoustic nerve (wave I) up to the lateral lemniscus (wave V). The central conduction time between these waves, along with IPL, are considered as indices of CNS functioning. The myelination of nerve fibers and maturation of synaptic relays are the reasons, as they have an exponential effect on the reduction of the conduction time of impulses [10,11].

The age distribution among the different groups varied significantly, with the SM group having the highest median age (34.5 years) (as shown in Table 1). This signifies that the participants enrolled in the study were young adults and, hence, the changes occurring due to aging were not present in any of them. This excludes the probability of prolonged latencies due to geriatric effects as reported by Konrad-Martin et al. [12], who stated that aging prolonged the latencies of early BAER waves due to a decreased number and/or synchrony of contributing auditory nerve units, hence prolonging the total conduction time of the impulses [12].

The median value for the duration of smoking was variable across the different subgroups (Table 1). The criterion of division of these groups was the HSI score of the participants; therefore, the duration of smoking was not considered an independent contributing factor, leading to the irregular findings. As anticipated, the median value of HSI scores was the highest (5.5) in the SH group.

The median values of latencies and IPL showed random variation across the four groups (Table 2 and Table 3) revealing no particular trend.

Progressing to the relationship between individual latencies (from wave I to wave V) and the HSI score of the participants (as indicated in Figure 3), the negative correlation of the latency of wave I implies that the speed of impulse transmission in the auditory nerve increases. This was explained by Gil and Metherate as the presence of nAChRs on the auditory nerve which are stimulated by the direct effect of nicotine via their alpha-7 subunits. These mediate the influx of calcium into the axon terminal leading to an increase in neurotransmitter release in the synaptic clefts, hence increasing the conduction velocity of the electrical signal. Furthermore, the presence of these receptors on the postsynaptic membrane leads to the increased excitability of the subsequent neuron, therefore decreasing the time required for the generation of the next impulse, even with sub-threshold stimuli, resulting in decreased latency [5]. Inconsistently, the negligible correlation of wave II latency with the HSI scores indicates that nicotine minimally affects the cochlear nuclei and that it acts as a link between the peripheral auditory nerve and the central nuclei present in the brainstem, which is inferred by the progressively increasing latencies at the level of the superior olivary nucleus (wave III), the lateral lemniscus (wave IV), and the inferior colliculus (wave V). This could be attributed to the chronic effects of hypoxia, hypercapnia, and neural injury caused by the toxic metabolites present in tobacco smoke, which may cause myelination defects, as stated by Kumar and Tandon [13].

The correlation of HSI scores with the IPL of BAER waves (represented by Figure 4) illustrates that the time taken for the neural impulse to travel from the auditory nerve to the central nuclei increases in proportion to nicotine dependence. It was noted that the pathway from the superior olivary nucleus to the inferior colliculus is minimally affected, therefore showing exemption from the aforementioned degenerative changes. As IPL are calculated after excluding the latency of wave I (the generation time of the impulse), they purely depict the time taken for the neural impulse to travel from the auditory nerve along the pathway. Consequently, the excitatory effect of nicotine is disregarded. The latencies of waves I-III showed the most prominent effects, that is, from the auditory nerve to the superior olivary nucleus.

Kayacan et al. [2], Kumar and Tandon [13], Atis et al. [3], and Gupta et al. [6] reported results consistent with our findings, documenting prolonged latencies of waves I, III, and V, signifying slower impulse conduction in the auditory nerve and pons. They attributed these changes to the effects of nicotine and toluene on myelination [13] and the degenerative effects of chronic hypoxia and hypercapnia on the neurons [3,6]. At the level of the pontomedullary junction in the brainstem, neuronal injury occurs due to the abovementioned factors, commensurate with nicotine dependence, which results in signal delays.

Proceeding to the correlation of the duration of smoking (in years) with the BAER wave latencies (msec) of waves I-V (depicted in Figure 5), the observed inverse statistical correlation implied that as the duration of exposure to tobacco smoke lengthens, the time required for signal initiation and transmission from the auditory nerve to the midbrain declines. The stated trend is understood through the previous explanation that nicotine acts on the pre- and postsynaptic receptors in the auditory pathway. Furthermore, the axonal receptors in the subcortical structures of the brain enhance the synchrony of axonal propagation. As insisted by Domino and Kishimoto [14], the "protective shield" or "stimulus barrier" to irrelevant stimuli was increased by interaction with tobacco smoke, signifying that meaningful stimuli are processed better and faster under the influence of nicotine. It was deduced that consumption of the same leads to "attentional narrowing" and an enhancement in the relay speed of these signals [14].

A noteworthy observation in the pattern of associations was that the degree of reciprocal correlation of latencies of each subsequent wave with the duration of smoking diminished progressively from wave I to wave V. The magnitude of nerve propagation time reduction in the auditory nerve was much greater than that in the inferior colliculus (which was almost negligible). This led us to postulate that the stimulatory effect of chronic nicotine contact manifests mainly in the lower-order neurons of the auditory pathway, indicating that the improvement in synaptic relay function is predominantly due to the impact on the peripheral neurons of the sensory circuits rather than the higher-order neurons.

The relationship of the IPL of the BAER waves (illustrated in Figure 6) with the duration of smoking (years) indicates that the transit time of a pre-existing impulse from the auditory nerve to the superior olivary nucleus increased non-significantly, while that of the inferior colliculus has increased significantly in relation to the length of the smoking period. This infers that prolonged tobacco smoke inhalation slowed the impulse conduction at the level of the medulla and pons, thus impairing the transmission in the brainstem. 

The statistical significance of interdependence of the HSI score was highest for the IPL I-V, while that of the duration of smoking was maximum for IPL III-V, followed by IPL I-V. It is therefore safe to assert that long-term interaction with large amounts of tobacco smoke leads to diminution in the conduction efficiency of neurons at the level of the midbrain. This observation is supported by the conclusions of Kayacan et al., who stated that the neurons' demyelinating injury was largely present in the higher-order neurons, impeding their relay functions [2].

The detail to grasp from the above data is that the latencies of individual waves became shorter with the lengthening of the smoking interval, whereas the IPL became longer, that is, the time required to produce the action potential shortens, while that for the impulse to be carried through the pathway increases. The stimulation caused by nicotine leads to the activation of the nAChRs, which causes easy neuronal excitation. Conversely, when this timespan is excluded, it becomes rather obvious that the transmission rate of impulses is significantly decreased. The impact of nicotine dependence and duration of smoking is minimal on IPL I-III. Hence, this leads to the assertion that the degenerative effects of persistent smoke inhalation were diminished on the peripheral neurons but much more pronounced on the brainstem.

Additionally, a two-way ANOVA (reported in Table 4) was calculated to find the variance in latencies of BAER waves I-V in the left and right ears. The distinction in the latencies of waves I, III, and IV between both ears signified that the impact of tobacco smoke on the left and right ears was substantially variable at the levels of the auditory nerve, the superior olivary nucleus, and the lateral lemniscus. This inference has followed the previously mentioned trend of affecting the upper medulla and the pons. It is proclaimed that these are also the areas of crossing over of neurons in the auditory pathway, and the prolonged latencies observed are in the same neurons. 

The computed variance of IPL I-III, I-V, and III-V between the left and right ears (shown in Table 5) indicated no relevant finding in any of the values.

The present study addresses the prevailing lacuna in understanding the implications of the extent and duration of tobacco smoke on the CNS. It was conducted using highly sophisticated and accurate equipment, making the results more reliable. The participants in this study were age-matched males from the same geographical area, which eliminated a substantial number of confounding factors, leading to more precise observations. All conclusions have been drawn after a thorough review of the literature. 

At last, we will end by mentioning the various limitations of our study. This study was conducted only on male participants. Hence, the effects of the same factors on females are yet to be assessed. The cause of the contradictory effects of nicotine on peripheral lower-order neurons and central higher-order neurons should be further researched in future studies. No literature is yet available to explain the variation in the effects of nicotine on both ears, which mandates its future investigation. BAERs can only map neuronal activity up to the level of the brainstem. The electrical activity mapping in the cerebral cortex needs more sensitive and advanced equipment. A potential research question for further exploration of the topic could be as follows: "Is the decreased impulse generation time in the auditory nerve due to the lingering impact of recent smoking, or could it be a result of the prolonged presence of nicotine in the body?."

## Conclusions

It was concluded that at the pontomedullary level of the brainstem (i.e., BAER waves III, IV, and V), nicotine, toluene, and other toxic metabolites, along with chronic hypoxia, hypercapnia, and respiratory acidosis, cause long-term myelination defects, leading to slower conduction velocities and increased latencies, thus impeding central neuronal processing. This effect is progressively and significantly linked to the degree of nicotine dependence (as calculated by the HSI score) as well as the duration of smoking (in years), proving that the observed changes are directly linked to tobacco smoke inhalation.
